# The effects of metformin on stereological and ultrastructural features of the ovary in streptozotocin -induced diabetes adult rats: An experimental study

**DOI:** 10.18502/ijrm.v13i8.7506

**Published:** 2020-08-19

**Authors:** Parisa Mehrabianfar, Farzaneh Dehghani, Nargess Karbalaei, Fakhroddin Mesbah

**Affiliations:** ^1^Department of Anatomical Sciences School of Medicine, Shiraz University of Medical Sciences, Shiraz, Iran.; ^2^Histomorphometry and Stereology Research Center, Shiraz University of Medical Science, Shiraz, Iran.

**Keywords:** Diabetes, Metformin, Ovary, Tissue.

## Abstract

**Background:**

Diabetes is a chronic disease that can affect almost all of the body organs, including male and female reproductive systems.

**Objective:**

This study was designed to investigate the preventive effects of metformin on stereological and ultrastructure characteristics of the ovary in the streptozotocin-induced diabetes adult female rats.

**Materials and Methods:**

Seventy adult (8-10 wk) female Sprague-Dawley rats (180-200 gr) were equally divided, as follows: (n = 10/each) control; STZ-induced diabetes (single dose of 65 mg/kg STZ, IP); metformin-treated (50 mg/100 gr of body weight, orally); diabetic-metformin-treated; sham 1, (single dose of sodium citrate); sham 2, (0.5 ml of daily oral distilled water); and sham 3, (sodium citrate + distilled water treated). The body mass index, ovarian weight, blood sugar level, cholesterol, and triglyceride were measured. The stereological and ultrastructural features of ovary were assessed.

**Results:**

The blood sugar of induced-diabetic rats was increased (p < 0.01). The BMI (p < 0.01), number of granulosa cells (p = 0.04), primordial, primary and secondary follicles (p = 0.03), total volume of ovary (p < 0.01) and cortex, nucleus diameter ratio to the ooplasm were decreased. The number of atretic follicles in the diabetic and diabetic + metformin-treated rats were increased (p < 0.01). The ultrastructural characteristics of ovary were more damaged in diabetic rats.

**Conclusion:**

Diabetes has destructive effects on ovarian follicles and causes follicular atresia. Also, the size of oocytes, numbers of granulosa cells and ooplasmic organelles, which are involved in the folliculogenesis are affected by diabetes and metformin has no preventive effects.

## 1. Introduction 

Diabetes is a common chronic disease which is mainly characterized by high blood glucose concentration. The growth in urbanization and lifestyle changes has led to a significant increase in the prevalence of the disease (1, 2). Diabetes can affect most organs of the body, particularly the circulatory, visual, renal, and the peripheral nervous systems, and the functioning of thyroid and suprarenal glands. It also affects both male and female reproductive systems (3).

Previous studies show that diabetes has negative effects on the growth of the ovarian follicles and decreases the rate of fertility, but the exact mechanisms are unclear (4, 5). Alterations of glucose concentration can greatly affect some functions of reproductive system (6). For example, in streptozotocin (STZ)-induced diabetic rats, high level of glucose reduces the production of progesterone and estradiol in granulosa cells (GC) and causes reproductive disorders due to changes in folliculogenesis and steroidogenesis (7). Given the rapid incidence of diabetes and increase in the number of women with diabetes, it is essential to develop the animal models of diabetes to understand the effects of diabetes on the ovary and to identify ovarian dysfunctions, as well as to achieve a new treatment approach (8).

STZ is a preferred chemical agent that can be used to create an animal model of diabetes (9). It is a glucosamine-nitrosourea compound that has specific toxic effects on pancreatic beta cells. The mechanism of diabetes mellitus in experimental animals is not entirely clear, but it seems that the production of free radicals such as superoxide-hydrogen, peroxide, and nitric oxide has destructive effects of STZ on DNA strand of pancreatic beta cells (10).

In recent years, significant progress has been made in understanding and controlling diabetes, and in many cases we have been able to delay or prevent the complications of the disease (11). Metformin is an insulin-sensitizing drug and helps ovulation. This drug can help regulate the sexual cycle in about half of women with polycystic ovary syndrome. It can also reduce insulin levels by lowering appetite and helping in weight loss. Regular use of metformin along with other therapeutic proceedings including exercise, diet, and weight control can be more effective (12). Despite the long-term experimental and clinical experience with metformin in animal models and human, the exact mechanism of its function is still unclear (13). However, metformin is still used in the clinic without a perfect understanding of its mechanism. Therefore, more research is needed to investigate the effect of metformin on the reproductive system of diabetic patients (14).

To distinguish ovarian dysfunction, the ethical restrictions of human studies lead us to use animal models to investigate the effect of diabetes on accurate morphological (ultrastructure) and exact morphometric (stereological) features of ovary. So, this study was designed to investigate the preventive and therapeutic roles of metformin on ultrastructural and stereological features of ovary in the STZ-induced diabetes adult female rats.

## 2. Materials and Methods

### Animals

#### Animal selection

Seventy female Sprague-Dawley rats aged 8 to 10 weeks with a weight of 180-200 gr were used in this experimental study.

### Experimental design 

The vaginal smear test was done to confirm the diestrus phase of estrous cycle. Rats were randomly and equally classified into seven groups (n = 10/each):

1. Control group, received no drugs.

2. Experimental-1 (STZ-induced diabetes) group, received a single dose of 65 mg/kg STZ in sodium citrate (SC) (29.41 mg/1 ml normal saline) intraperitoneal (IP) on the first day of the first week.

3. Sham-1 (SC-treated) group, received SC, the same as the experimental-1 group.

4. Experimental-2 (Metformin-treated) group, received 50 mg/100 gr of body weight daily per os in 0.5 mL distilled water (DW) on the first day of the fifth week for three weeks (15).

5. Sham-2 (DW-treated) group, received DW, the same as the experimental-2 group.

6. Experimental-3 (STZ-induced diabetic + Metformin-treated) group, the induced-diabetic rats received metformin the same as the experimental-2 group.

7. Sham-3 (SC & DW-treated) group, received SC and DW, the same as the experimental-1 and 2 groups, respectively.

### Induction of diabetes

Diabetes was induced by a single IP injection of 65 mg/kg STZ (Sigma Aldrich, Germany), 15 min after the IP administration of 110 mg/kg of nicotinamide (Ranbaxy Chemicals Ltd., Mumbai, India). STZ was dissolved in SC buffer (pH = 4.5) and nicotinamide in 0.9% normal saline (16). To confirm hyperglycemia, the glucose level in plasma was measured by the glucometer strips (Easy gluco-Auto-Coding) at 10 days after the STZ injection. The threshold value of plasma glucose to diagnose diabetes was taken as > 200 mg/dL.

### Measurement of the body weight, height, and body mass index (BMI)

To calculate the BMI, the body weight and height of the rats (from nose to root of tail) were measured using the digital scale and digital caliper with 0.01 precision, respectively, once a week for 8 weeks on the same day at the same time.

### Measurement of blood sugar levels 

The blood sugar levels were measured using a glucometer on 1${}^{\mathrm{st}}$, 10${}^{\mathrm{th}}$, and 28${}^{\mathrm{th}}$ day to confirm diabetes. Animals with blood glucose levels > 200 mg/dL after 10 days were confirmed as diabetic (16).

### Measurement of blood level of cholesterol and triglyceride

The serum levels of cholesterol and triglyceride were assessed once on the 28${}^{\mathrm{th}}$ day after the administration of STZ injection in all rats using ELISA kit (United States, Abcam).

### Measurement of ovary weight

After sacrificing the rats, removal and trimming of the ovaries, the weight of right ovaries was measured by digital scale (ScalTec, spo51, Germany) with 0.01 precision.

### Stereological study

The right ovary of each animal was dissected and trimmed, and then fixed with 10% formalin for several days. The tissue-processing method was applied to prepare the paraffin embedding blocks. The blocks were cut into 5 μm and 25 μm serial thick sections, stained with Hematoxylin & Eosin (H & E), and observed by a light microscope. Eight to twelve sections from each ovary were selected by the systematic random sampling, then the stereological software designed at the Shiraz University of Medical Sciences, Shiraz, Iran, the stereological probes (point grids and counting frames) were superimposed onto the video images of the tissue sections and viewed on the monitor.

### Estimation of the number of granulose cells and follicles

**Figure 1:**
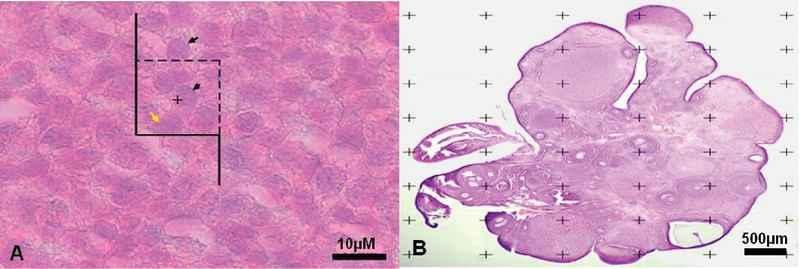
A: The optical dissector method. The numerical density of granulosa cells using the unbiased counting frame. The black arrows indicate the cells to be counted and the yellow arrow shows the uncounted cell. B: Point counting method to estimate the volume density of the cortex, medulla, and the total volume of the ovary.

We used a Nikon E200 microscope (Nikon, Tokyo, Japan) which was fitted at the 60x oil immersion objective. The fields were selected through the systematic random sampling. Also, a microcator that was mounted on the microscope was used to measure the movement along the z-axis (Figure 1A). Finally, the following formula was used for estimating the number of cells. 

$$N_{V(\mathrm{cells})}=\{\sum Q/\left(a/f\times \sum P\times h\right)\times t/BA$$

### Total volume of the ovary, cortex, and medulla

The Cavalieri method was used to count the total number of points superimposed on the images (point-counting method) (Figure 1B), and the following formulas were applied: 

$$V_{\left(\mathrm{ovary}\right)}=\sum p\times a\left(p\right)\times T$$

V( cortex , medulla )=∑P cortex , medulla /∑P ovary

Using stereological software, the diameters of the nucleus and the cytoplasm were measured in oocytes.

### Tissue preparation for transmission electron microscopy

The left ovaries were trimmed and immersed in 2.5% glutaraldehyde for 2-3 hr and then segmented into 1 mm 3 dimensions. The segments were primary-fixed in 2.5% glutaraldehyde overnight. Then, the samples were washed three times with 0.1 M sodium cacodylate buffer and post-fixed in 1% buffered osmium tetroxide for 1.5 hr. After fixation, the samples were washed three times with 0.1 M sodium cacodylate buffer and two times with DW. The segments were followed by dehydration in gradually increasing graded series of 30-100% ethanol and infiltration by different ratios; 1:3, 1:1, 3:1 of resin/ethanol and then in pure resin overnight. The samples were embedded in resin (Agar 100). The blocks were polymerized at 60°C overnight. The semi-thin sections (1 μm thickness) were prepared with glass knife and stained with 1% toluidine blue and overviewed by light microscope. The ultra-thin sections (60-90 nm thickness) were prepared with diamond knife, transferred onto the 200mesh grids and stained with uranyl acetate and lead citrate. The samples were observed by TEM (Philips CM10, Amsterdam, Netherlands).

### Ethical consideration

The animals were housed under controlled conditions (12:12 hr light-dark cycle at 23 ± 2°C), with ad libitum access to food and tap water for 8 wk. Also, animal handling and all other procedures were done following the ethical principles and the national norms and standards for conducting medical research in Iran, which was approved by the Ethics committee of Shiraz University of Medical Sciences, Shiraz, Iran (Approval ID: IR.SUMS.REC.1397.659).

### Statistical analysis

We used commercial scientific statistic software; graph pad prism version 7.05, (Prism Inc, San Diego, California, USA) in order to analyze my scientific data and present graph, Data are reported as means and standard deviation and analyzed using One-way ANOVA test. In addition, for statistical comparisons between the groups, we used Tukey's multiple comparisons test as a post-hoc test. The mean difference was considered significant if p < 0.05.

## 3. Results

### Body mass index 

**Figure 2:**
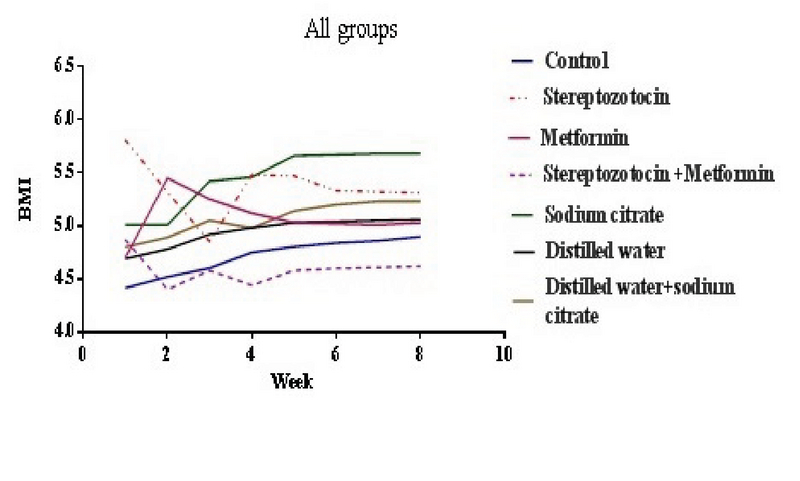
Mean of BMI in all of the groups. STZ-induced diabetic (experimental-1) + Metformin-treated (experimental-2) vs DW and SC (sham-3) (p = 0.03) in the second week.

The BMI was significantly reduced in the experimental-1 and experimental-3 groups in the second week of experiment (p < 0.01, p = 0.03 respectively) (Figure 2).

### Blood sugar levels

Mean ± SD of blood sugar levels of animals in the experimental-1 (p < 0.01) and experimental-3 groups (p < 0.01) showed a significant increase compared to the sham-1 and sham-3 groups on 10${}^{\mathrm{th}}$ day of experiment respectively. Also in the experimental-1 and experimental-3 rats, a significant increase was seen compared to the control group (p < 0.01 and p < 0.01, respectively).

In addition, on the 28th day of experiment, blood sugar was significantly higher in the the experimental-1 (p = 0.01) and experimental-3 groups (p = 0.01) compared to the control group.

There were no significant differences in the blood sugar levels of animals between groups on the first day of experiment (Table I).

### The serum levels of cholesterol and triglyceride

There were no significant differences in the serum levels of cholesterol and triglyceride between the groups (Table II).

### Weight of ovaries

Despite the results of this study that show 18% decrease in the ovarian weight in experimental-1 group and 30% increase in ovarian weight in experimental-3 group compared to the control group, there were no significant differences in the ovarian weight between the studied groups.

### Stereological study

#### Estimation of the number of GCs

**Figure 3:**
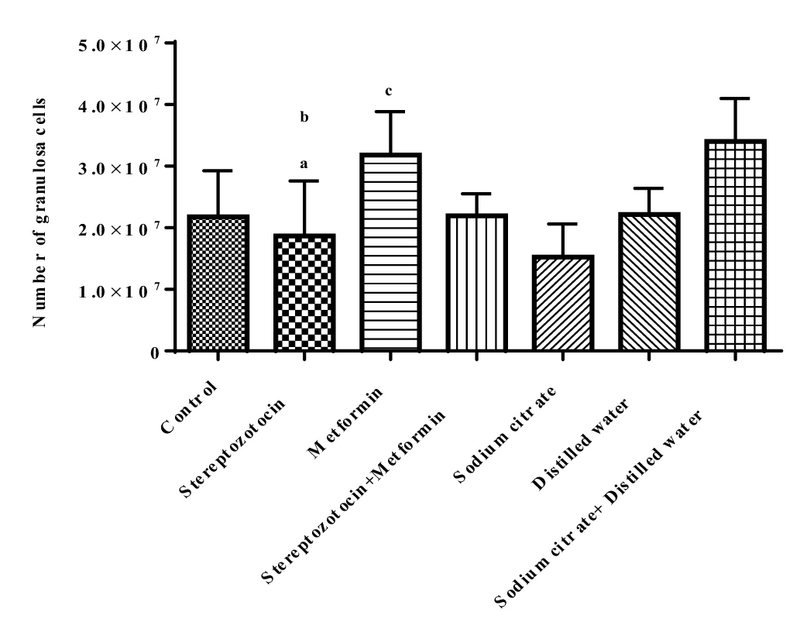
Mean ± SD of the number of granulosa cells. (a) Streptozotocin vs metformin (p = 0.04). (b) Streptozotocin vs sodium citrate and distilled water (p = 0.01). (c) Metformin vs sodium citrate (p = 0.01).

The mean ± SD of the number of GC was significantly decreased (p = 0.04) in the experimental-1 group as compared with the experimental-2 group and also sham-3 group (p = 0.01). In addition, there was a significant increase in experimental-2 group compared to the sham-1 group (p = 0.01; Figure 3).

#### Number of follicles 

The results of this study indicated that the mean ± SD of the number of primordial follicles were significantly decreased in the experimental-1 group compared with experimental-2 (p = 0.03), and the primary follicles were significantly decreased (p < 0.01) in the experimental-1 groups as compared with the experimental-2 and sham-3 group (p = 0.04). The secondary follicles were significantly decreased (p = 0.01) in the experimental-1 as compared with the experimental-2 group.

No significant differences were observed between the mean ± SD of the numbers of antral follicle in all of the groups. The mean ± SD of the number of atretic follicles in the experimental-3 rats was significantly higher than the other rats (p < 0.01) (Table III).

#### Total volume of the ovary, cortex, and medulla 

The mean ± SD of the total volume of ovary was significantly increased in experimental-2 group compared to the experimental-1 (p < 0.01) and control groups (p = 0.01). Also, in the total volume of ovary, a significant increase was seen in the experimental-3 group as compared with the experiment-1 (p = 0.01).

**Figure 4:**
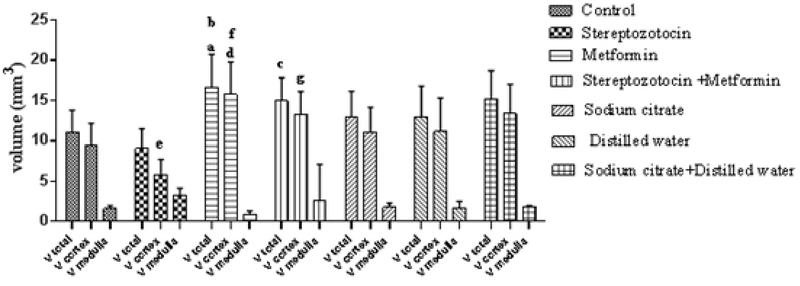
The comparison of the total volume of ovary, cortex, and medulla in all of the groups, a: metformin vs control (p = 0.01), b: metformin vs STZ (p < 0.01), c: STZ + metformin vs STZ (p = 0.01), d: metformin vs control (p < 0.01), e: STZ vs sodium citrate (p = 0.01), f: metformin vs STZ (p < 0.01), and g: STZ + metformin vs STZ (p < 0.01).

The mean ± SD of the volume of cortex significantly increased in the experimental-2 group compared to the control group (p < 0.01). Further, in experimental-1 rats, a significant decrease in the volume of cortex was seen compared to the sham-1 group (p = 0.01). Besides, the volume of cortex was significantly increased in the experimental-2 group than the experimental-1 group (p < 0.01). Moreover, there was a significant increase in experimental-3 group compared to the experimental-1 group (p < 0.01). There were no significant differences in the mean ± SD of the volume of medulla between all of the groups (Figure 4).

#### Ratio of the diameter of nucleus to ooplasm

**Figure 5:**
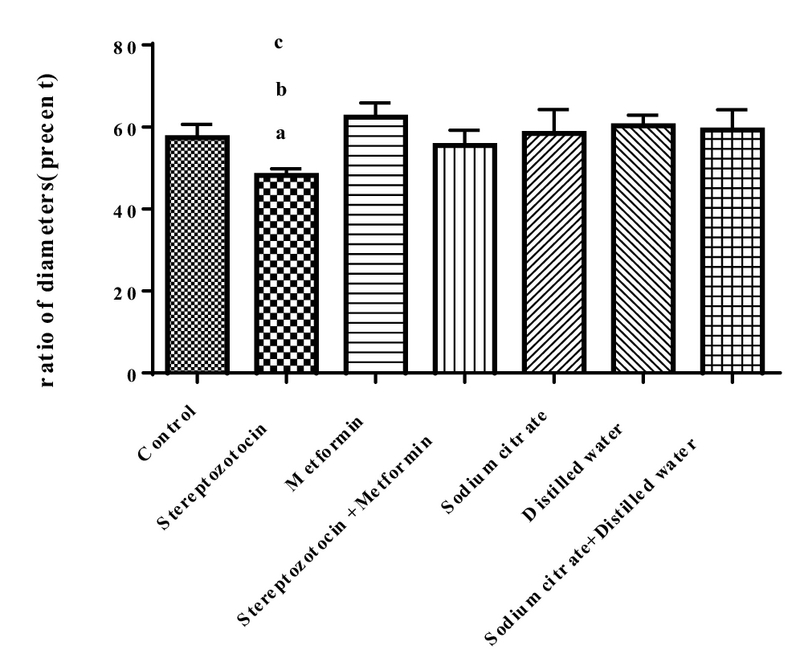
The ratio of the diameter of the nucleus to the cytoplasm of the oocyte in all of the groups. a: streptozotocin vs control (p < 0.01), b: streptozotocin vs metformin (p = 0.05), and c: streptozotocin vs sodium citrate (p < 0.01).

The mean ± SD of the ratio of the diameter of the nucleus to the ooplasm was decreased significantly in the experimental-1 group compared to the control (p < 0.01), and experimental-2 (p = 0.05), and sham-1 groups (p < 0.01) (Figure 5).

### Histological observation of resin embedded sections 

#### Control group

The germinal epithelium (GE) that contained cuboidal cells with distinct boundaries and underlying tunica albuginea (TA) were observed. The ovarian stroma contains normal cells and blood vessels. Some primordial and primary follicles with normal GCs with dividing nuclei and oocyte with typical distribution of organelles in the cortex were observed. Outside the basement membrane, theca interna, and externa cells with long and spindle shape were seen. The thickness of zona pellucida (ZP) was normal and perivitelline space (PVS) wasn't observed (the figure is not shown).

#### Experimental groups

#### Experimental-1

**Figure 6:**
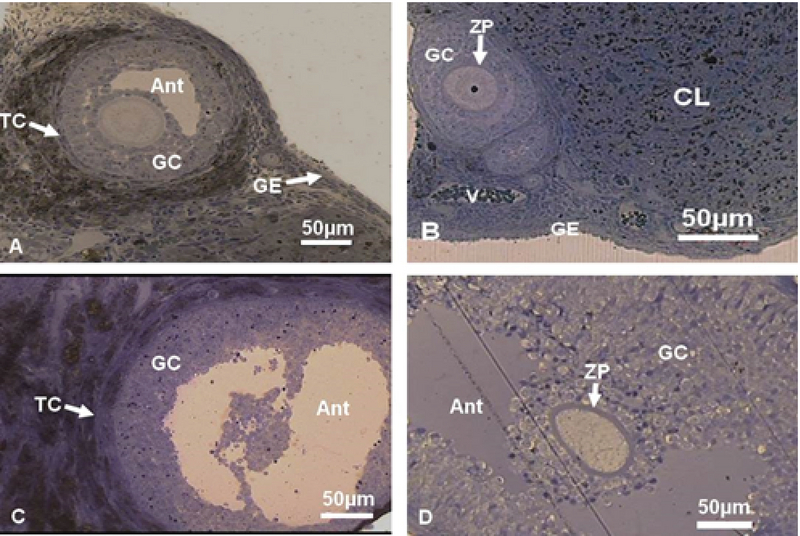
Light micrograph of ovarian tissue of resin-embedded sections (Toluidine blue staining). (A) Diabetic metformin-treated. (B) Sodium citrate + distilled water-treated group. (C) STZ-induced diabetic. (D) Metformin-treated. Ant, Antrum; CL, Corpus lueum; GC, Granulosa cells; TC, Theca cells; V, Vessele; ZP, Zona pellucida; and GE, Germinal epithelium.

The ovaries of diabetic rats were covered by squamous epithelium and the TA was unclear. The number of degenerative oocytes increased. The cytoplasm of the GCs and theca cells (TCs) contained many vacuoles (VC). There were many degenerated GCs and TCs with pyknotic nuclei. The ZP had normal thickness and the ooplasm had homogenous distribution of organelles (Figure 6).

#### Experimental-2 

The intact GE with cuboidal and squamous cells and distinct boundaries and underlying TA was observed. The GCs and TCs and also stromal cells were full of VC. The ZP with regular thickness and normal distribution of ooplasmic organelles were observed (Figure 6).

#### Experimental-3 

The GE had cuboidal and also squamous cells. The underlying TA was unclear. The GCs had normal appearance but there were some degenerated cells. The TCs with foamy cytoplasm were observed and also there was no clear boundary between interna and externa. The ZP had regular thickness. The normal distribution of ooplasmic organelles was observed. Some primordial follicles with normal GCs and normal oocytes were seen (Figure 6).

#### Sham groups

#### Sham-1

Germinal epithelium with normal and cuboidal cells were observed. A number of follicles were seen within the cortex. The ovarian stromal cells were normal. There were no pyknotic nuclei in GCs. The ooplasmic organelles had regular distribution (the figure is not shown).

#### Sham-2

The cuboidal and squamous cells were observed in GE. The underlying TA was distinct. Some of the stromal cells contained VC. The GCs were completely normal without the pyknotic nuclei. The ZP with regular thickness was observed. The normal ooplasmic distribution of organelles was detected.

#### Sham-3

The cuboidal and columnar epithelial cells covered the ovaries and also stromal cells had normal appearance. The blood vessels that contained RBC were noticed in ovary cortex. The primordial, primary and antral follicles with normal GCs and regular ZP thickness were observed (Figure 6).

### Ultrastructural observation

#### Control group

**Figure 7:**
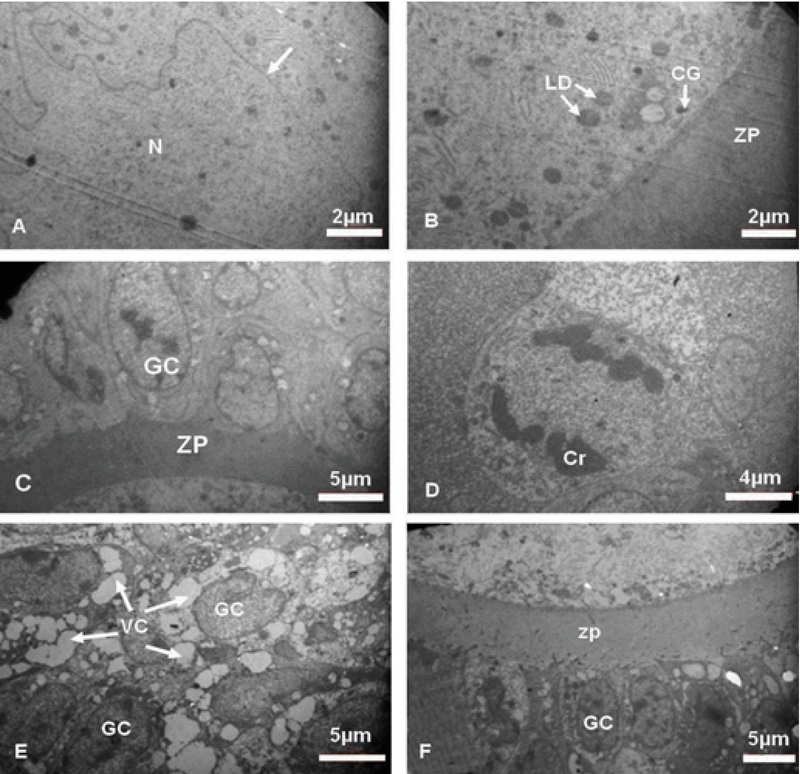
Electron micrograph of ovarian tissue. (A, B) Control (unnamed white arrow shows nuclear envelope), (C, D) Diabetic, (E, F) Metformin-treated groups. Cr, Chromosome; GC, Granulosa cells; LD, Lipid droplet; N, Nucleous; VC, Vacuoles; ZP, Zona pellucida.

The GCs were normal with distinct cytoplasmic membrane. The normal and regular distribution of ooplasmic organelles such as endoplasmic reticulum and mitochondria (Mt) were observed. The ZP had regular thickness and contained microvilli. There were some cortical granules (CGs) and lipid droplet (LD) in close contact with oolemma. The double membrane of nucleus with nuclear envelope pores and reticular nucleolus were distinct (Figure 7).

#### Experimental groups

#### Experimental-1

The nuclei of the GCs were observed with the dense chromatin. The cytoplasm of TCs and GCs contained many VCs. The GCs degenerated and TCs became inflamed with foamy cytoplasm. In spite of the large number of degenerated GCs, a number of mitotic cells were also observed. The irregular ZP with less thickness was observed and did not contain microvilli and cumulus cells process endings. The oolemma was not clearly defined and perivitelline space was not seen. The distribution of oocyte organelles was uniform. The two-layer membrane of the nuclear envelope with its pores and reticular nucleolus was observed in the oocyte (Figure 7).

#### Experimental-2

The ovaries of metformin-treated rats contained GCs with pyknotic nuclei and cytoplasmic VC. The TCs layers were normal with some VC. The ZP with normal thickness was observed and no perivitelline space was seen. The ooplasmic organelles had regular distribution. Also, in the ooplasm a few Mt and annulate lamella were observed (Figure 7).

#### Experimental-3

The few GCs were destroyed; however, the basement membrane remained intact and separated the TI and GCs layers. There was some VC in GCs. The cytoplasmic organelles of GCs, especially Mt, had regular distribution. The cell junctions between the GCs were not observed.

**Figure 8:**
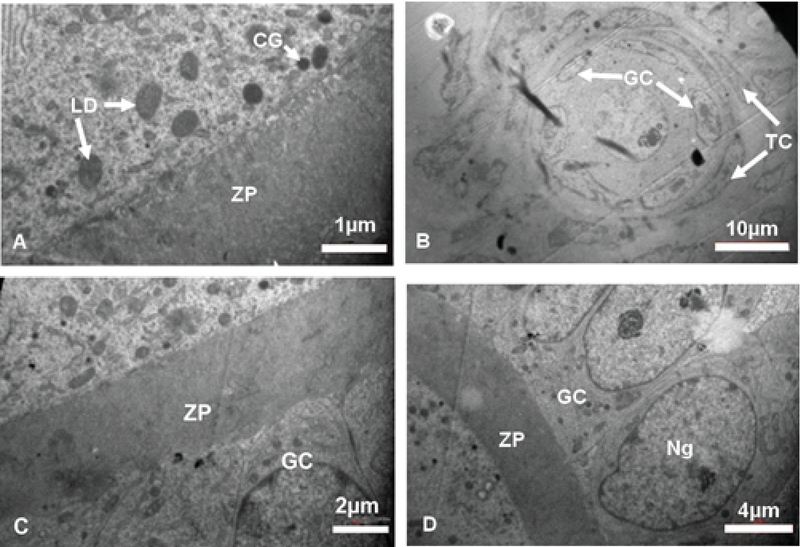
Electron micrograph of ovarian tissue. (A, B) Diabetic metformin-treated, (C, D) Sodium citrate + distilled water groups. CG, Cortical granule; GC, Granulosa cells; LD, Lipid droplet; Ng, Nucleus of granulosa cells; ZP, Zona pellucida.

The ZP had regular thickness and contained microvilli. The organelles were scattered throughout ooplasm. Some LDs and CGs were in close contact with oolemma. A lot of AL was seen in ooplasm. The double layer of nuclear envelope with nuclear pores was noticed. A primordial follicle with normal oocyte and normal distribution of ooplasmic organelles and also normal GCs and TCs were observed (Figure 8).

#### Sham groups

#### Sham-1

Most GCs were normal and the TI and granulose layer were also separated by intact basement membrane. The few TCs were destroyed. The ZP had regular thickness. Some ooplasmic organelles like AL and Mt with regular distribution were observed. There were some CGs and LDs under the oolemma (not shown here).

#### Sham-2

The GCs and TCs were normal. The ZP had regular thickness and the ooplasmic organelles were distributed regularly. There were some CGs and LDs in close contact with the oolemma (not shown here).

#### Sham-3

The GCs were columnar and distinct membrane and cell junctions were noticed. Also, the GCs with distinct nucleolus and nuclear membrane were seen. ZP had regular thickness. The CGs were seen under oolemma. The theca layers with spindle-shaped cells were seen outside of the GCs (Figure 8).

**Table 1:** Mean ± SD of blood sugar level (mg/dL) on 1${}^{\mathrm{st}}$, 10${}^{\mathrm{th}}$, and 28${}^{\mathrm{th}}$ day


**Groups/days**	**1${}^{\mathrm{st}}$ day**	**10${}^{\mathrm{th}}$ day**	**28${}^{\mathrm{th}}$ day**
**Control**	95.00 ± 8.33	97.60 ± 3.50	94.20 ± 12.74
**Experimental-1 **	81.60 ± 29.18	329.0 ± 106.60${}^{*}$	345.0 ± 80.64${}^{***}$
**Experimental-2 **	96.00 ± 11.02	99.60 ± 11.84	98.40 ± 8.503
**Experimental-3 **	102.8 ± 21.44	229.8 ± 48.45${}^{**}$	229.4 ± 48.11${}^{****}$
**Sham-1 **	98.00 ± 7.71	105.0 ± 7.61	99.60 ± 4.722
**Sham-2 **	103.2 ± 6.76	115.0 ± 14.58	106.2 ± 10.38
**Sham-3 **	103.8 ± 19.41	103.0 ± 11.47	94.40 ± 12.40
* Experimental-1 vs. Control (p < 0.01); ** Experimental-3 vs. Control (p < 0.01); *** Experimental-1 vs. Control (p = 0.01); **** Experimental-3 vs. control (p = 0.01)			

**Table 2:** Mean ± SD of The serum levels of cholesterol and triglyceride


**Groups/cholesterol and triglyceride**	**Cholesterol**	**Triglyceride**
**Control**	54.6 ± 9.94	185.6 ± 69.11
**Experimental-1**	49.8 ± 8.28	159 ± 58.24
**Experimental-2**	58.6 ± 11.3	156.3 ± 44.71
**Experimental-3**	60.8 ± 9.576	211.5 ± 42.29
**Sham-1**	48.2 ± 13.81	176 ± 81.36
**Sham-2**	56.6 ± 13.58	144 ± 48.99
**Sham-3**	49.2 ± 2.588	89.5 ± 7.141

**Table 3:** Mean ± SD of the number of follicle in all of the groups


**Groups/Follicles**	**Primordial**	**Primary**	**Secondary**	**Antral**	**Atretic**
**Control**	256.2 ± 230.9	634.3 ± 411	969.4 ± 191.3	815.3 ± 402.8	150.3 ± 101.2
**Experimental-1**	112.7 ± 83.29${}^{a}$	229 ± 150.9${}^{b}$	550.8 ± 190.0c	700.0 ± 192.2	221.5 ± 112.4
**Experimental-2**	540.0 ± 371.3	975.7 ± 280.9	1232 ± 369.7	1210 ± 593.4	279.7 ± 62.48
**Experimental-3**	299.1 ± 120.6	590.4 ± 225.9	870.4 ± 259.5	795.4 ± 334.2	733.8 ± 152.7${}^{d}$
**Sham-1**	128.0 ± 32.02	279.5 ± 156.3	1034 ± 199.2	329.9 ± 147.5	218.7 ± 82.93
**Sham-2**	287.3 ± 129.2	598.6 ± 232.1	991.8 ± 291.1	1025 ± 242.9	146.6 ± 61.31
**Sham-3**	334.6 ± 243.6	795.8 ± 372.1	1044 ± 306.0	347.2 ± 155.3	147.0± 54.83
${}^{a}$ Experimental-1 vs. Experimental-2 (p = 0.03); ${}^{b}$ Experimental-1 vs. Experimental-2 (p < 0.01) and Sham-1 (p = 0.04); ${}^{c}$ Experimental-1 vs. Experimental-2 (p = 0.01); ${}^{d}$ Experimental-3 vs. Other groups (p < 0.01);					

## 4. Discussion 

The results of our study show that, STZ induces the appropriate diabetic rat model; it is confirmed by high levels of blood sugar and reduction of body weight. STZ-induced diabetes decreases the number of dynamic follicles and increases atretic follicles, and has destructive effects on ovarian tissue. Therefore, the weight and volume of ovary is reduced. Also, the numbers of GCs and crucial organelles of oocyte and GCs, which are involved in the growth and development of the follicles are affected by diabetes.

The results of present study show a significant BMI reduction in STZ-induced diabetic rats, same as the body weight reduction in patient with diabetes (17). This is confirmed by the animal model studies that show a reduction in the body weight of the STZ-induced diabetic rats (18). In spite of this, after about three weeks, the BMI reduction remains constant until the end of the experiment, which is similar to the result of Courteix and Eschaliera (19). In our study, the metformin also cannot inhibit the weight decline in STZ-induced diabetic rats.

One of the important indexes in the evaluation of ovarian functions is the change in the weight and volume of ovary, which is the result of alteration in ovarian structures, like the change in the number and nature of follicles. In this study, there is a rational reduction in the weight of ovary in STZ-induced diabetic rats and also a non-statistically significant weight gains in STZ-induced diabetic + metformin-treated rats. Tesone and colleagues observed the ovarian weight reduction in diabetic animal model (20). Their results indicate that ovarian tissue is damaged by diabetes and metformin has somewhat prevented this impairment.

The size and volume of ovary affect the ovarian functions and the destructive effects of diabetes on the growth and development of follicle can reduce the volume of cortex and subsequently the total volume of ovary. In this study, the cortical and total volume of ovary decreased in STZ-induced diabetic rats and metformin was partly able to recover this reduction; on the other hand, in the non-diabetic rats, metformin also increased the volume of ovaries. A clinical study showed that 25% of the ovarian volume decreased in diabetic patients per year versus 13% in healthy people (21). The reduction of cortical volume in diabetic rats indicates damage to the cortex and reduces the number of follicles and GCs. An estimation of the exact number of ovarian follicles at different stages of development is an important index of folliculogenesis and shows the maturation of oocyte and supporting cells. In this research, the number of granulose cells and of course the primordial, primary and secondary, but not antral, follicles were reduced and atretic follicles in diabetic rats increased, so the diabetes can increase the degeneration of GCs and follicular atresia. In 2005, a study on STZ-diabetic model reported that hyperglycemia decreases the expression of connexin 43. This reduction can affect the communication between the oocyte and GCs, so the degeneration of GCs increased, also the reduction of connexin43 expression causes abnormal oocyte and weak folliculogenesis (4). The number of GCs increased in metformin-treated rats, but there was no difference between the STZ-induced diabetic + metformin-treated and STZ-induced diabetic rats without any treatment, so that, metformin cannot prevent the reduction of GCs in diabetes.

The supportive roles of GCs is established through the gap junction between GCs and oocytes, which is important to transport the essential nutrients to the oocyte (22). Therefore, due to damage of GCs in STZ-induced diabetic rats, it is not unexpected that the damage in primordial, primary and secondary, but not antral follicles will increase, because these types of follicles need GCs for growth and development process. So, in this research there was no significant difference in the number of antral follicles between the STZ-induced diabetic and control groups. Similar conclusions were obtained by Erbas and colleagues in 2014; this study was conducted in diabetic rats and showed that diabetes affects ovarian follicles, especially in primordial, primary and secondary follicles, but does not affect the number of antral follicles. They believed that the cause of the increased follicular atresia is a result of damage to the GCs (23), which may be due to the less biochemical needs of the oocytes.

The growth and development of an oocyte simultaneously requires both nuclear and cytoplasmic maturation. Although, oocytes with nuclear maturation can be fertilized, they may fail due to the deficiency of cytoplasmic factors that are essential to complete the maturation process (24).

The reduction in the ratio of the diameter of the nucleus to the cytoplasm can be caused by the reduction of oocyte diameter in diabetic rats. Previous research on STZ-induced diabetic models suggests that the size of oocytes in the antral follicle is less than other nondiabetic groups (4). Also, Afrough and colleagues reported that diabetes causes a delay in the arrival of oocyte into metaphase II and decreases oocyte diameter and increases the perivitelline space (25). The results further show that the ratio of the diameter of the nucleus to the ooplasm increases significantly in the metformin-treated compared to the STZ-induced diabetic rats. Metformin has not been able to recover this reduction in the STZ-induced diabetic rats, because the results indicate that there is no significant difference in the STZ-induced diabetic + metformin-treated rats compared to the STZ-induced diabetic rats.

Both the histological and ultrastructural aspects of ovary in this study revealed serious injury in the follicles of STZ-induced diabetic rats. These are in agreement with the morphometric finding, because the results of this study show that the dynamic types of follicles were reduced and degenerated follicles and oocytes increased. Nevertheless, as a result of this damage and the reduction in the number of supportive cells, intercellular junction between the GCs and oocyte was not clearly observed. The supportive roles of GCs and cell junctions between GCs and oocytes are of particular importance for the transmitting of growth factors and nutrient materials for cytoplasmic and nuclear maturation. Even bidirectional communication between GCs and oocytes is essential for cumulus cells expansion, as the third aspect of follicular development (26). In STZ-induced diabetic rats, the TCs were not spindle shaped and became swollen. It is well-known that the endocrine roles of the TCs are essential for the production of estrogen, the function of TCs is like a double-edged blade, both over and under activity of these cells lead to infertility due to hyperandrogenism and lack of estrogen, respectively (27). We also observed the irregular thicknesses of ZP in STZ-induced diabetic rats. The ZP ensures the major roles in fertilization and communication between oocytes, and also as a non-cellular structure, it has a protective role for oocytes, eggs, and embryos (28). The thickness of ZP seems to be a major factor in sperm penetration. In the clinic, a thick ZP (≥ 22 µm) is used as a major indicator in the microinjection procedure (29). Generally, it can be concluded that the diabetes has introduced the irreversible damages to ovarian tissue, which can cause infertility.

Also, ultrastructural observations on the ovary of non-diabetic metformin-treated rats showed degeneration and the large numbers of VC in the cytoplasm and of GCs, while the stereological study did not show any reductions in the number of GCs. The VC can be residues of the LDs that are used to produce energy for normal cell activity (30). However, there were no VCs in the STZ-induced diabetic + metformin-treated rats and also the degeneration of GCs was decreased. This study has shown that metformin in non-diabetic rats has a deleterious effect on ovarian structures. Previous studies have shown that metformin does not affect ovarian functions in human with normal or low levels of testosterone, while in patients with high testosterone levels, metformin increases the ovulation (31). Previous research has shown that testosterone levels in diabetics are reduced (32), as we find in our study (not shown in the result section).

## 5. Conclusion

STZ induces the appropriate diabetic rat model; it is confirmed by high levels of blood sugar on the 10th day of injection. The STZ-induced diabetes reduces body and ovarian weights, decreases the number of dynamic follicles and increases atretic follicle, and has destructive effects on ovarian tissue. Therefore, the total and cortical volume of ovary is reduced. Also, the fertility criteria such as the size of follicle and oocyte, numbers of GCs and crucial organelles of oocyte and GCs, which are involved in the growth and development of the follicles are affected by diabetes. Hence, it seems that diabetes can lead to reduced fertility and metformin has no preventive effects.

##  Conflict of Interest

The authors declare no conflict of interest.
